# Efficacy and safety of oral ivermectin versus benzyl benzoate for the treatment of scabies: a systematic review and meta-analysis of randomized controlled trials

**DOI:** 10.3389/fmed.2025.1703912

**Published:** 2025-11-06

**Authors:** Ahmed Abu-Zaid, Hoor Ahmad AlBdah, Latifa AlKandari, Retaj S. Aljuma, Shaikha T. H. Alhussaini, Hawraa Yaqoub Alqallaf, Fai M. Alsaleeli, Rashed Ahmed Soud Alhusaini, Danah S. Alrasheedi, Jumanah Abdulrahman Alshammari, Ali Ashkanani, Abdullah M. Alharran

**Affiliations:** 1 Department of Biochemistry and Molecular Medicine, College of Medicine, Alfaisal University, Riyadh, Saudi Arabia; 2 Kuwait Institute for Medical Specializations, Kuwait City, Kuwait; 3 College of Medicine and Medical Sciences, Arabian Gulf University, Manama, Bahrain; 4 Ministry of Health, Kuwait City, Kuwait

**Keywords:** itch, scabies, ivermectin, benzyl benzoate, meta-analysis

## Abstract

**Background/objectives:**

Oral ivermectin and topical benzyl benzoate are two common treatment options for scabies, but there is ongoing discussion regarding their relative safety and efficacy. A thorough synthesis of the available evidence is required to inform treatment decisions because of the clinical debate caused by the contradictory findings from current randomized controlled trials (RCTs).

**Methods:**

A systematic review and meta-analysis were conducted on evidence retrieved from PubMed, Scopus, Web of Science, and CENTRAL for RCTs up to August 2025. The primary outcome was the cure rate. Secondary outcomes included pruritus improvement and the incidence of adverse events. Stata MP v. 18 was used to pool outcomes.

**Results:**

Ten RCTs involving 1,105 patients were included. Cure rates showed no significant difference between ivermectin and benzyl benzoate at 1 week (RR: 1.07, 95% CI [0.88, 1.30], *p* = 0.51), 2–4 weeks (RR: 0.99, 95% CI [0.88, 1.12], *p* = 0.91), or after more than 4 weeks (RR: 1.16, 95% CI [0.95, 1.43], *p* = 0.15). The overall pooled result confirmed no difference (RR: 1.04, 95% CI [0.95, 1.14], *p* = 0.37). For pruritus, no significant differences were observed at 1 week (RR: 1.07, 95% CI [0.80, 1.43], *p* = 0.66), 2–4 weeks (RR: 1.19, 95% CI [0.97, 1.46], *p* = 0.09), or beyond 4 weeks (RR: 1.10, 95% CI [0.89, 1.37], *p* = 0.38); overall RR: 1.13, 95% CI [0.99, 1.29], *p* = 0.07. Ivermectin showed significantly fewer adverse events (RR: 0.27, 95% CI [0.16, 0.46], *p* < 0.001), particularly less burning/stinging (RR: 0.07, 95% CI [0.02, 0.20], *p* < 0.001). Gastrointestinal (GI) events were not significantly different (RR: 1.47, 95% CI [0.67, 3.22], *p* = 0.34).

**Conclusion:**

Oral ivermectin and topical benzyl benzoate exhibit comparable efficacy for the treatment of scabies. However, ivermectin’s significantly better safety and tolerability, combined with the practical advantage of oral administration, establish it as a valuable and often preferable therapeutic choice.

**Systematic review registration:**

CRD420251143937.

## Introduction

With estimates from the World Health Organization (WHO) showing a prevalence of over 200 million people affected at any given time and an annual incidence of over 400 million cases, scabies poses a significant global public health burden (1). Scabies was officially designated a Neglected Tropical Disease (NTD) by the WHO in 2017 (2), acknowledging its significant impact. Scabies results from a skin infestation by the microscopic mite *Sarcoptes scabiei* var. hominis, which burrows into the epidermis and causes intense, unrelenting itching (3). This intense itching frequently causes scratching, potentially leading to secondary bacterial infections from pathogens such as *Staphylococcus aureus* and *Streptococcus pyogenes* (4), leading to severe complications, including impetigo, cellulitis, sepsis, and post-streptococcal diseases such as acute glomerulonephritis and potentially rheumatic heart disease (5). Resource-limited communities are disproportionately affected by scabies, with overcrowding and poverty contributing to its transmission (6).

Scabies management primarily involves topical scabicides and oral ivermectin (7). Although 5% permethrin is frequently advised as a first-line treatment, increasing reports of treatment failure have prompted interest in effective alternatives (8), bringing renewed focus to two widely used treatments: oral ivermectin and topical benzyl benzoate. Ivermectin is typically praised for its simple administration as a one-time oral dose, notably boosting patient compliance and positioning it as a prime agent for large-scale drug administration efforts intended to control community outbreaks (9). Conversely, benzyl benzoate presents a well-established and economical topical alternative, thereby ensuring accessibility in numerous resource-constrained settings (10). Its utility is, however, potentially restricted by a rigorous application protocol and the capacity to induce local cutaneous irritation, manifesting as burning and stinging sensations (11). A randomized controlled trial (RCT) has determined benzyl benzoate to be superior (6). At the same time, other RCTs have found ivermectin to be more effective (12, 13), and several others have reported no significant difference in efficacy between the two treatments (11, 14).

Because of this discrepancy in the literature, currently, there is a substantial knowledge gap that prevents clinicians from reaching a clear consensus regarding the relative safety and effectiveness of topical benzyl benzoate versus oral ivermectin in the treatment of scabies. Therefore, we conducted this systematic review and meta-analysis of RCTs to comprehensively compare the efficacy, safety, and tolerability of oral ivermectin versus topical benzyl benzoate for the treatment of scabies.

## Methods

### Protocol registration

This systematic review was registered with the International Prospective Register of Systematic Reviews (PROSPERO) with CRD420251143937. Furthermore, the methodology for this systematic review and meta-analysis followed the Preferred Reporting Items for Systematic Reviews and Meta-Analyses (PRISMA) statement (15) and the Cochrane Handbook for Systematic Reviews of Interventions (16).

### Data sources and search strategy

On 7 August 2025, a literature search was systematically conducted across several electronic databases, including PubMed, Scopus, Web of Science, CENTRAL, and Google Scholar. The search strategy utilized a combination of the following keywords: (Scabies OR “Norwegian Itch” OR “Sarcoptes scabiei” OR “Sarcoptic Mange”) AND (ivermectin OR Eqvalan OR Ivomec OR MK-933 OR MK933 OR “MK 933” OR Mectizan OR Stromectol) AND (“benzyl benzoate” OR “BB” OR Benzanil OR Novoscabin OR Ansar OR Antiscabiosum OR Ascabiol OR Benzemul OR Acarosan OR Acaril).” The search was conducted without any limits, except for Scopus, where the search was limited to titles, abstracts, and keywords. A detailed breakdown of the search terms and results for each database is provided in Supplementary Table S1. Moreover, to ensure thoroughness and avoid overlooking any relevant studies, a manual search of reference lists from relevant trials was performed.

### Eligibility criteria

RCTs adhering to the following Population, Intervention, Control, and Outcome (PI-CO) framework were eligible for inclusion: Population (P): Patients with a clinical or parasitological diagnosis of scabies, regardless of disease severity. Intervention (I): oral ivermectin, regardless of the dosing regimen or any co-administered drugs. Control (C): benzyl benzoate, regardless of the dosing regimen or any co-administered drugs. Outcomes (O): The primary outcome was the cure rate, defined as the absence of lesions or negative parasitological examination. Secondary outcomes included improvement in pruritus and safety outcomes, including the incidence of any adverse events, such as burning/irritation or any gastrointestinal adverse event. Studies were excluded based on the following criteria: quasi-randomization; investigation of combined scabies treatment protocols; publication as conference abstracts or proceedings; or study designs as observational studies, *in vitro* studies, or reviews.

### Study selection

The screening and selection of studies were independently conducted by two reviewers using Covidence software. Following the automated removal of duplicate entries, the remaining unique articles were subjected to a two-phase screening process. Titles and abstracts were initially screened, followed by a full-text assessment of potentially eligible studies. Disagreements among the reviewers were resolved through discussion, culminating in a consensus.

### Data extraction

An Excel spreadsheet was created for data extraction purposes and was piloted using the full texts of the articles included. The extraction form was organized into three main sections: (A) Study Characteristics: study ID, country, study design, total number of patients, treatment protocols, cure rate definition, method of scabies diagnosis, primary outcome assessment tool, key inclusion criteria, and follow-up duration. (B) Participant baseline characteristics: age, gender, and family history. (C) Outcome Data: cure rate, improvement in pruritus, any adverse events, burning/irritation, and any gastrointestinal adverse event.

The data were independently extracted by two reviewers. All discrepancies were resolved through discussion and consultation with a senior author. For dichotomous data, event numbers and the total number of participants were extracted. For continuous data, means and standard deviations were extracted. We utilized the formulas proposed by Wan et al. (17) to convert data presented as median and interquartile range or range into mean and standard deviation.

### Risk of bias and certainty of evidence

The methodological quality and risk of bias for each included RCT were assessed using the revised Cochrane Collaboration’s Risk of Bias tool (ROB 2) (18). Two reviewers independently evaluated each study across domains such as selection bias, performance bias, reporting bias, and attrition bias. Disagreements were resolved by consensus. Additionally, the overall certainty of the evidence was assessed using the Grading of Recommendations Assessment, Development, and Evaluation (GRADE) approach (19, 20). This framework considers factors such as the risk of bias, inconsistency, indirectness, imprecision, and publication bias. Each factor was carefully assessed, and the rationale for each judgment was documented, with any discrepancies resolved through discussion.

### Statistical analysis

All statistical analyses were performed using Stata MP version 18 (Stata Corp.). The risk ratio (RR) was calculated for dichotomous outcomes, and the mean difference (MD) was used for continuous outcomes, both presented with their 95% confidence intervals (CI). The standardized mean difference (SMD) was utilized when studies measured the same continuous outcome on different scales. A fixed-effects model was the default model for analysis; however, a random-effects model was employed if substantial heterogeneity was present. Heterogeneity was evaluated using the chi-squared (χ^2^) test and the I^2^ statistic. A *p*-value of less than 0.1 for the χ^2^ test or an I^2^ value of 50% or higher was indicated significant heterogeneity. An assessment of publication bias was not performed, as all analyzed outcomes included fewer than 10 RCTs (21). Where data permitted, subgroup analyses were conducted at various time points based on the number of oral ivermectin doses (single vs. double) and the concentration of benzyl benzoate (<25% vs. ≥25%).

## Results

### Search results and study selection

**Figure 1:**
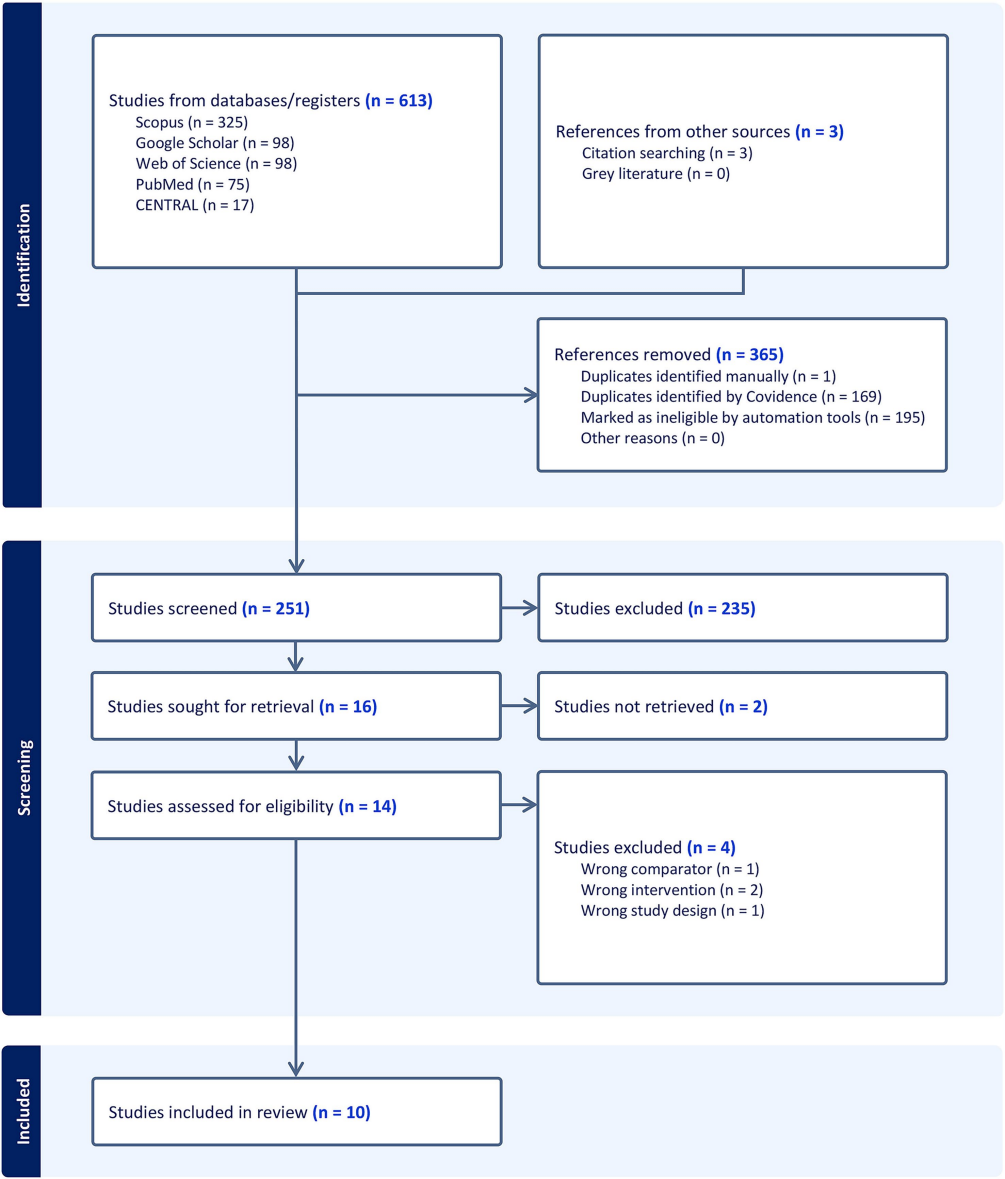
PRISMA flowchart of the screening process.

The initial literature search yielded 613 records, and 3 records were added by citation searching. After 365 duplicates were automatically removed, the titles and abstracts of the remaining 251 articles were screened. This led to the exclusion of 235 studies that did not meet the inclusion criteria. Consequently, 16 articles were assessed for eligibility via full-text screening. Of these, four studies were excluded for different reasons (Supplementary Table S2). Ultimately, 10 RCTs (4, 6, 10–14, 22–24) were included in the qualitative and quantitative synthesis (Figure 1).

### Characteristics of included studies

Ten RCTs and 1,105 patients were included in our pooled analysis (4, 6, 10–14, 22–24). All RCTs investigated oral ivermectin versus topical benzyl benzoate, with various treatment protocols. All included trials were open-label, except for Brooks et al., which was an observer-blinded RCT (11). Most trials provided adjuvant drugs, which commonly included antihistamines for pruritus and antibiotics for secondary bacterial infections. The ivermectin group consisted of 531 patients, and the benzyl benzoate group consisted of 574 patients. Full details about the study characteristics and patients’ baseline data are available in Tables 1, 2.

**Table 1:** Summary characteristics of the included RCTs.

Study ID	Country	n	Ivermectin details	Benzyl benzoate details	Adjuvant drugs	Method of diagnosis	Main inclusion criteria	Cure rate definition	Primary outcome	Follow-up duration
Abdel-Raheem et al. (4)	Egypt	100	Dosage: 200 μg/kg; Schedule: Two doses, 1 week apart; Route: Oral; Instructions: Taken with meals	Concentration: 20%; Formulation: Cream; Schedule: Five consecutive nights; Contact Time: Left overnight	Antihistamines for cured participants. Azithromycin for secondary infections pre-treatment	Clinical presentation and parasitological examination (skin scrapings)	Ages 5–50 years, weight >15 kg, with ordinary scabies	Negative parasitological examination with a complete absence of new lesions	Cure rate and adverse drug reaction monitoring	2 weeks
Babu et al. (22)	India	130	Dosage: 200 μg/kg; Schedule: Single dose; Route: Oral; Instructions: Not specified	Concentration: 25%; Formulation: Lotion; Schedule: Single application; Contact Time: 24 h	Oral hydroxyzine (10 mg or 25 mg) was given for symptomatic management of pruritus	Clinically diagnosed	Ages 5–60 years, patients willing to receive either oral or topical therapy	Improvement in the severity of pruritus and lesions	Improvement in clinical grading of disease (%) and pruritus (%)	6 weeks
Bachewar et al. (10)	India	52	Dosage: 200 μg/kg; Schedule: Single dose; repeated after 1 week if no signs of cure; Route: Oral; Instructions: not specified	Concentration: 25%; Formulation: Lotion; Schedule: Two consecutive nights; repeated after 1 week if no improvement; Contact Time: Left overnight	Antihistamines for cured participants. Antibiotics (azithromycin or ampicillin) for secondary infection pre-treatment	Diagnosis was based on clinical symptoms and history	Ages >12 years, newly diagnosed patients of scabies	Absence of any new lesions (papules, vesicles, or burrows)	Cure rate, adverse drug reaction (ADR) monitoring	2 weeks
Brooks et al. (11)	Vanuatu	80	Dosage: 200 μg/kg; Schedule: Single dose; Route: Oral; Instructions: Directly observed treatment	Concentration: 10%; Formulation: Topical mixture; Schedule: Single application; Contact Time: Applied at night	Antibiotics were administered for bacterial superinfection	Diagnosed based on a consistent history and typical lesions	Ages 6 months to 15 years	No skin lesions noted at 3 weeks post-treatment	Number of scabies lesions, itch visual analogue score, and nocturnal itch	3 weeks
Chitra et al. (23)	India	100	Dosage: 200 μg/kg; Schedule: Single dose; Route: Oral; Instructions: On an empty stomach	Concentration: 25%; Formulation: Lotion; Schedule: Single application; Contact Time: 24 h	Family members and close contacts were issued 25% BB lotion. Antipruritic medicines were prohibited	Diagnosed clinically based on nocturnal itching and typical scabietic lesions	Ages 5–60 years with uncomplicated scabies	Subsidence of lesions and itching	Efficacy (subsidence of lesion and itching) and safety	4 weeks
Ly et al. (6)	Senegal	181	Dosage: 150–200 μg/kg; Schedule: Single dose; repeated at day 14 if failed; Route: Oral; Instructions: On an empty stomach.	Concentration: 12.5%; Formulation: Not specified; Schedule: One group had a single application; another had two applications 24 h apart; Contact Time: 24 h per application	Oral antibiotics (amoxicillin or erythromycin) for superinfection pre-randomization	Clinical criteria and parasitological examination (skin scrapings)	Ages 5–65 years with characteristic lesions and itching	The complete disappearance of visible lesions and itching	Disappearance of skin lesions and itching at day 14	4 weeks
Mallya et al. (12)	India	60	Dosage: 200 μg/kg; Schedule: Two doses, 10 days apart; Route: Oral; Instructions: Taken with meals	Concentration: 25%; Formulation: Lotion; Schedule: Three consecutive nights; Contact Time: At least 8 h	Use of antipruritic agents was prohibited	Clinical diagnosis and microscopic demonstration of the mite	Ages 5–60 years, newly diagnosed patients of scabies	No new lesions, improvement in pruritus, and negative parasitological examination	Therapeutic efficacy (clinical and pruritus scores) and cost-effectiveness	3 weeks
Manjhi et al. et al. (24)	India	120	Dosage: 200 μg/kg; Schedule: Single dose; Route: Oral; Instructions: Not specified	Concentration: 25%; Formulation: Lotion; Schedule: Single application; Contact Time: Left overnight	NR	Clinically diagnosed patients	Ages 5–60 years, patients willing to undergo either topical or oral therapy	Improvement in the severity of pruritus and disease (lesions)	Improvement in the severity of disease and the severity of pruritus	6 weeks
Meyersburg et al. (14)	Austria	224	Dosage: 200 μg/kg; Schedule: Two doses, 1 week apart; Route: Oral; Instructions: Not specifically advised to take on an empty stomach.	Concentration: 25% (10% for children 1–5 years); Formulation: Emulsion; Schedule: Daily for three consecutive days; Contact Time: Not washed off before midday of the fourth day	NR	Confirmed by dermoscopic detection of mites	Ages >1 year or weight > = 15 kg with dermoscopy-verified scabies	Absence of mites on dermoscopic examination	Comparative efficacy, safety, and tolerability	3 weeks
Nnoruka et al. (13)	Nigeria	58	Dosage: 200 μg/kg; Schedule: Single dose; Route: Oral; Instructions: Not specified	Concentration: 25%; Formulation: Emulsion; Schedule: Single application; Contact Time: 72 h	NR	Clinical criteria and confirmation with skin scrapings	Ages >5 years	Complete disappearance of initial skin lesions and pruritus	Efficacy and safety	4 weeks

RCT, randomized controlled trial; NR, not reported; BB, benzyl benzoate.

**Table 2:** Baseline characteristics of the participants.

Study identifier	Number of patients in each group		Age (years), Mean (SD)		Gender (male), N. (%)	
	Ivermectin	BB	Ivermectin	BB	Ivermectin	BB
Abdel-Raheem et al. (4)	50	50	27.84 (9.46)	22.52 (12.77)	26 (52)	24 (48)
Babu et al. (22)	65	65	26.18 (9.04)	27.12 (10.28)	NR	NR
Bachewar et al. (10)	27	25	NR	NR	14 (51.8)	18 (72)
Brooks et al. (11)	43	37	5.1 (3.9)	4.7 (3.8)	NR	NR
Chitra et al. (23)	50	50	NR	NR	NR	NR
Ly et al. (6)	65	116	61.5% ≤ 15 yrs	60.3% ≤ 15 yrs	45 (69.2%)	71 (61.2)
Mallya et al. (12)	30	30	Overall Mean: 23.7		NR	NR
Manjhi et al. (24)	60	60	NR	NR	NR	NR
Meyersburg et al. (14)	112	112	24.6 (14.5)	26.1 (18.9)	65 (58)	53 (47)
Nnoruka et al. (13)	29	29	Overall Mean: 27.9		Overall 35 Male; 33 Female	

NR, not reported; BB, benzyl benzoate; N., number of patients; SD, standard deviation.

### Risk of bias and certainty of evidence

**Figure 2:**
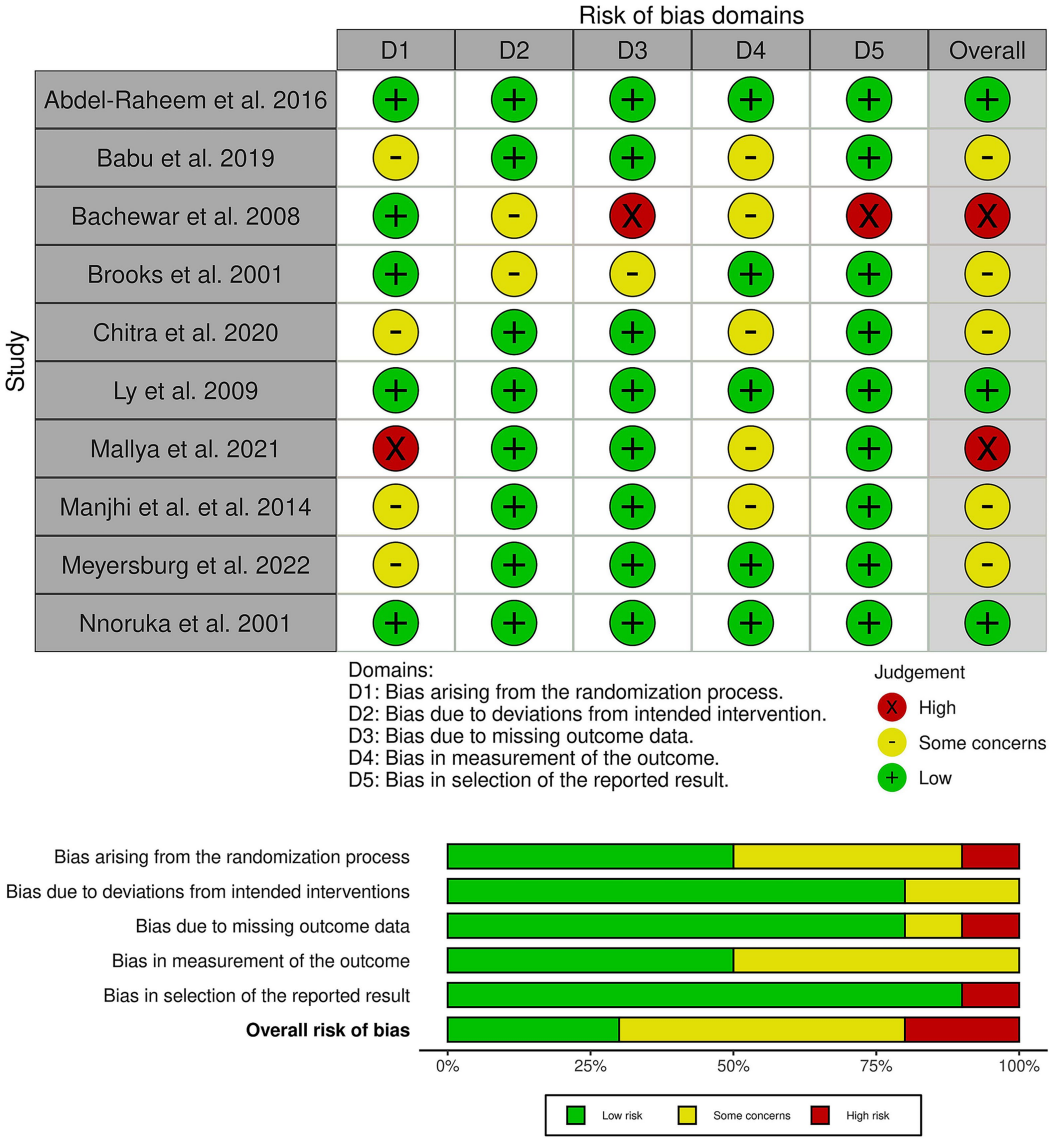
Quality assessment of risk of bias in the included trials. The upper panel presents a schematic representation of risks (low = green, unclear = yellow, and high = red) for specific types of biases of the studies in the review. The lower panel presents risks (low = red, unclear = yellow, and high = red) for the subtypes of biases of the combination of studies included in this review.

Three trials showed a low risk of bias (4, 6, 13), five trials showed some concerns of bias (11, 14, 22–24), and two trials had an overall high risk of bias (10, 12) (Figure 2). Brooks et al. raised concerns about attrition bias, as 30 of 110 patients (27%) were lost to follow-up without a clear rationale, and the trial employed a per-protocol analysis (11). Furthermore, Bachewar et al. (10) showed a high risk of attrition bias due to high and differential drop-out rates and the use of a per-protocol analysis. Finally, several trials showed a high risk of performance and detection bias due to the open-label design combined with subjective outcomes, such as itching scores and clinical lesion assessment, by unblinded investigators. Finally, Mallya et al. (12) showed a high risk of selection bias as the study is described as a quasi-experimental study, which contradicts its claim of using computer-generated random numbers.

Furthermore, the outcome-based certainty of evidence assessment is described in detail in Table 3.

**Table 3:** GRADE evidence profile of certainty of evidence.

Certainty assessment							Summary of findings				
Participants (studies) follow-up	Risk of bias	Inconsistency	Indirectness	Imprecision	Publication bias	Overall certainty of evidence	Study event rates (%)		Relative effect (95% CI)	Anticipated absolute effects	
							With [Benzyl benzoate]	With [Ivermectin]		Risk with [Benzyl benzoate]	Risk difference with [ivermectin]
Cure rate after 1 week											
560 (6 RCTs)	Serious^a^	Not serious	Not serious	Serious^b^^,^^c^	None	⨁⨁◯◯ Low^a^^,^^b^^,^^c^	128/279 (45.9%)	141/281 (50.2%)	RR 1.07 (0.88 to 1.30)	128/279 (45.9%)	32 more per 1,000 (from 55 fewer to 138 more)
Cure rate after 2–4 weeks											
771 (7 RCTs)	Serious^a^	Not serious	Not serious	Not serious	None	⨁⨁⨁◯ Moderate^a^	338/410 (82.4%)	284/361 (78.7%)	RR 0.99 (0.88 to 1.12)	338/410 (82.4%)	8 fewer per 1,000 (from 99 fewer to 99 more)
Cure rate after >4 weeks											
308 (3 RCTs)	Serious^a^	Not serious	Not serious	Serious^b^^,^^c^	None	⨁⨁◯◯ Low^a^^,^^b^^,^^c^	96/154 (62.3%)	124/154 (80.5%)	RR 1.16 (0.95 to 1.43)	96/154 (62.3%)	100 more per 1,000 (from 31 fewer to 268 more)
Pruritis improvement after 1 Week											
350 (3 RCTs)	Serious^a^	Not serious	Not serious	Serious^b^^,^^c^	None	⨁⨁◯◯ Low^a^^,^^b^^,^^c^	58/175 (33.1%)	64/175 (36.6%)	RR 1.07 (0.80 to 1.43)	58/175 (33.1%)	23 more per 1,000 (from 66 fewer to 143 more)
Pruritis improvement after 2–4 Weeks											
318 (4 RCTs)	Serious^a^	Not serious	Not serious	Serious^b^^,^^c^	None	⨁⨁◯◯ Low^a^^,^^b^^,^^c^	94/155 (60.6%)	131/163 (80.4%)	RR 1.19 (0.97 to 1.46)	94/155 (60.6%)	115 more per 1,000 (from 18 fewer to 279 more)
Pruritis improvement after >4 Week											
250 (2 RCTs)	Serious^a^	not serious	not serious	very serious^b^^,^^c^	none	⨁◯◯◯ Very low^a^^,^^b^^,^^c^	87/125 (69.6%)	103/125 (82.4%)	RR 1.10 (0.89 to 1.37)	87/125 (69.6%)	70 more per 1,000 (from 77 fewer to 258 more)
Any adverse event											
755 (7 RCTs)	Serious^a^	Not serious	Not serious	Not serious	None	⨁⨁⨁◯ Moderate^a^	75/399 (18.8%)	14/356 (3.9%)	RR 0.27 (0.16 to 0.46)	75/399 (18.8%)	137 fewer per 1,000 (from 158 fewer to 102 fewer)
Burning/irritation											
755 (7 RCTs)	Serious^a^	Not serious	Not serious	Not serious	None	⨁⨁⨁◯ Moderate^a^	−/399	−/356	RR 0.07 (0.02 to 0.20)	−/399	0 fewer per 1,000 (from 0 fewer to 0 fewer)
Any gastrointestinal adverse event											
755 (7 RCTs)	Serious^a^	Not serious	Not serious	Very serious^b^^,^^c^	None	⨁◯◯◯ Very low^a^^,^^b^^,^^c^	0/399 (0.0%)	7/356 (2.0%)	RR 1.47 (0.67 to 3.22)	0/399 (0.0%)	0 fewer per 1,000 (from 0 fewer to 0 fewer)

CI, confidence interval; RR, risk ratio.

a Most trials showed either some concerns or a high risk of bias.

b A wide confidence interval that does not exclude the appreciable harm/benefit.

c Low number of events.

### Primary outcome: cure rate

**Figure 3:**
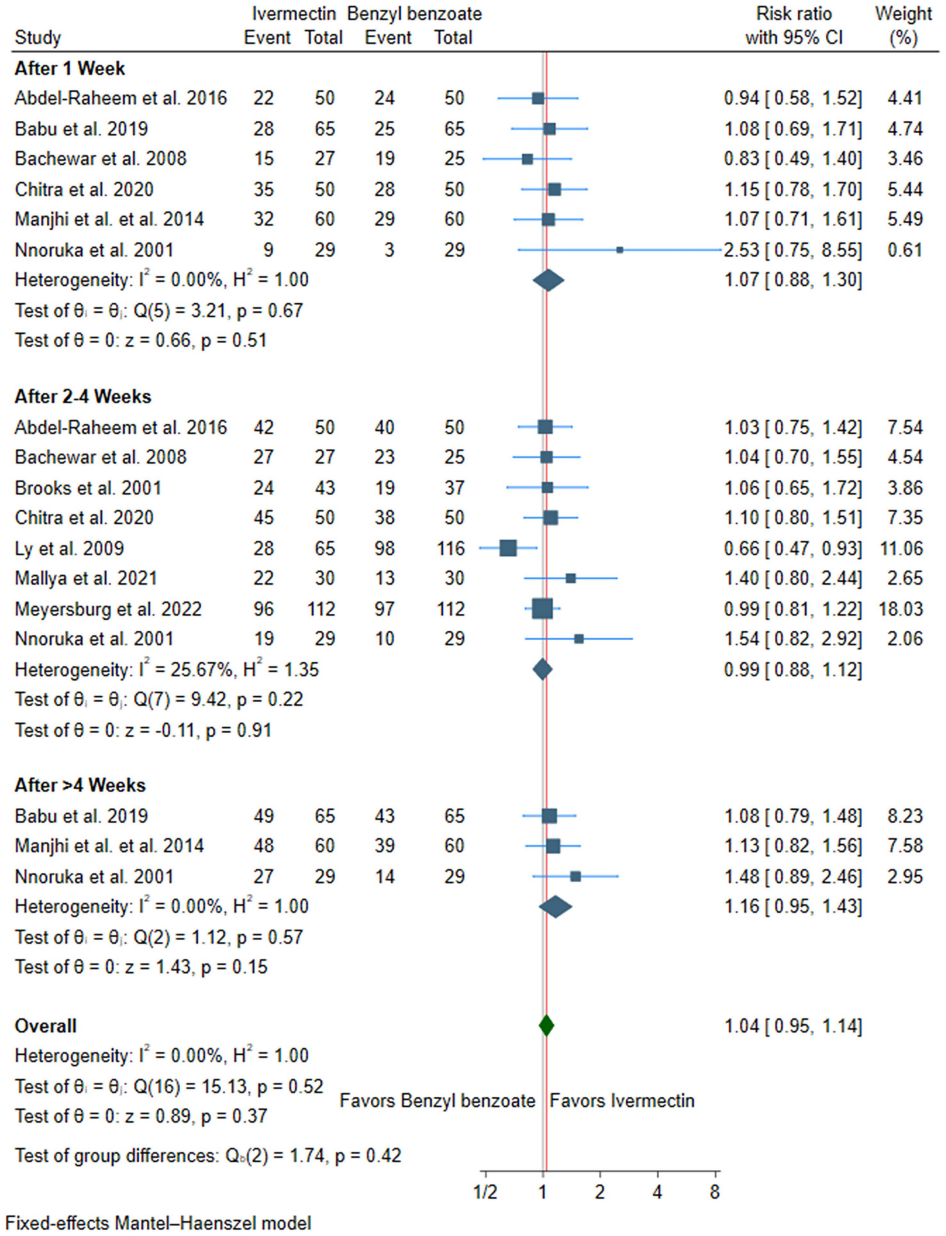
Forest plot of the primary outcome (cure rate). RR, risk ratio, CI, confidence interval.

There was no difference between ivermectin or benzyl benzoate after 1 week (RR: 1.07, with 95% CI [0.88, 1.30], *p* = 0.51, I^2^ = 0%), after 2–4 weeks (RR: 0.99, with 95% CI [0.88, 1.12], *p* = 0.91, I^2^ = 26), or after more than 4 weeks of follow-up (RR: 1.16, with 95% CI [0.95, 1.43], *p* = 0.15, I^2^ = 0%). Furthermore, the overall pooled analysis across all time points showed no significant difference between the two treatments (RR: 1.04, with 95% CI [0.95, 1.14], *p* = 0.37, I^2^ = 0%) (Figure 3).

Subgroup analysis according to the dose of ivermectin showed no significant difference between single and double doses, whether at 1 week or 2–4 weeks (Supplementary Table S3). Similarly, subgroup analysis by benzyl benzoate concentration revealed no significant difference between lower (<25%) and higher (≥25%) concentrations, whether at 1 week or 2–4 weeks (Supplementary Table S4).

### Secondary outcomes

#### Pruritus improvement

**Figure 4:**
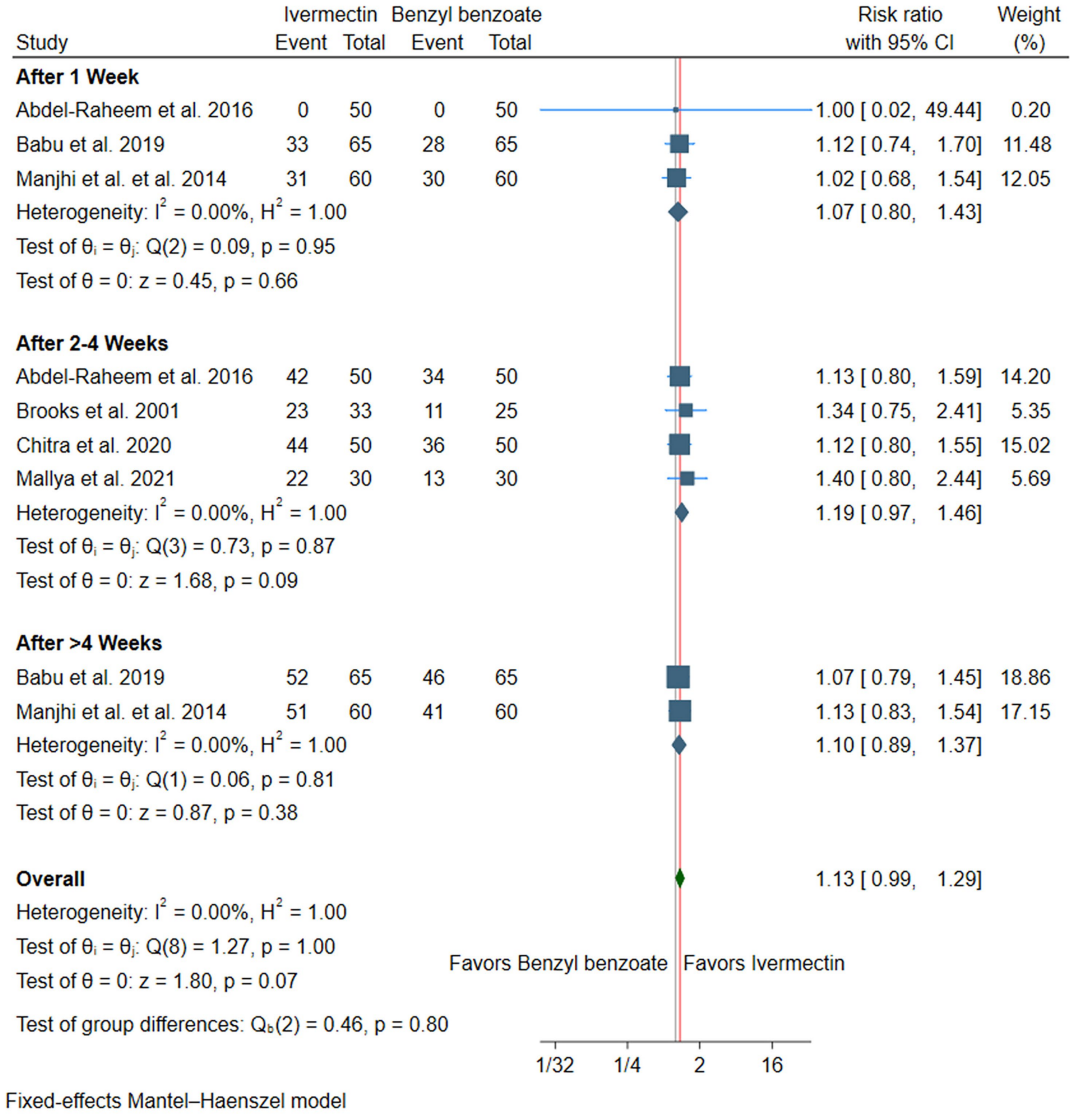
Forest plot of the secondary efficacy outcome (pruritus improvement), RR, risk ratio, CI, confidence interval.

There was no difference between ivermectin or benzyl benzoate after 1 week (RR: 1.07, with 95% CI [0.80, 1.43], *p* = 0.66, I^2^ = 0%), after 2–4 weeks (RR: 1.19, with 95% CI [0.97, 1.46], *p* = 0.09, I^2^ = 0%), or after more than 4 weeks of follow-up (RR: 1.10, with 95% CI [0.89, 1.37], *p* = 0.38, I^2^ = 0%). The overall pooled analysis across all time points also showed no significant difference between the two treatments (RR: 1.13, with 95% CI [0.99, 1.29], *p* = 0.07, I^2^ = 0%) (Figure 4).

Subgroup analysis by the dose of ivermectin at 2–4 weeks. Both subgroups showed statistically significant improvement in pruritus compared to benzyl benzoate. The single-dose subgroup demonstrated an RR of 1.27 (95% CI [1.04, 1.56], *p* = 0.02, I^2^ = 5%), while the double-dose subgroup showed an RR of 1.36 (95% CI [1.01, 1.83], *p* = 0.04, I^2^ = 36%) (Supplementary Table S3). Furthermore, subgroup analysis by the benzyl benzoate concentration at 2–4 weeks showed that oral ivermectin showed a statistically significant superiority when compared with benzyl benzoate at concentrations <25% (RR = 1.29, 95% CI [1.05, 1.58], p = 0.02, I^2^ = 0%). However, when the comparator was benzyl benzoate at concentrations ≥25%, the difference was not statistically significant (RR = 1.35, 95% CI [0.99, 1.86], *p* = 0.06, I^2^ = 45%) (Supplementary Table S4).

#### Safety outcomes

**Figure 5:**
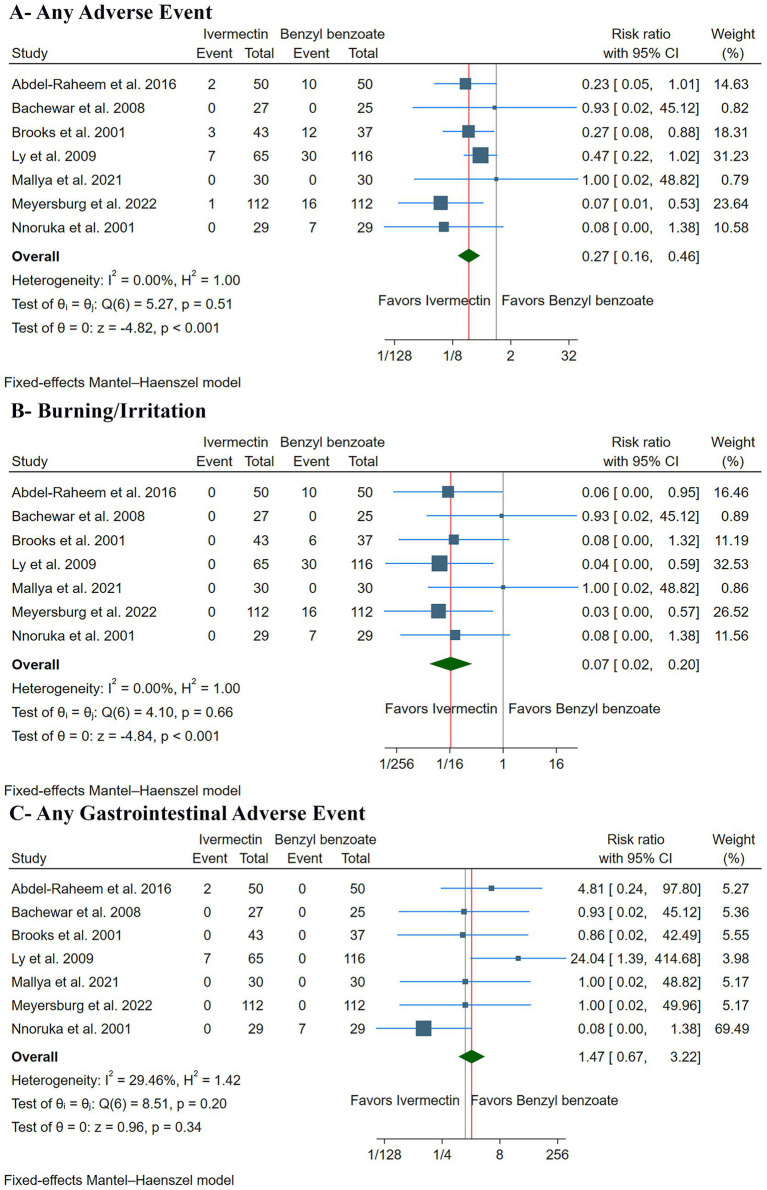
Forest plots of the secondary safety outcomes: **(A)** any adverse event, **(B)** burning/irritation, and **(C)** any gastrointestinal adverse event. RR: risk ratio, CI: confidence interval.

The pooled analysis showed that ivermectin was associated with a significantly lower risk of overall adverse events compared to benzyl benzoate (RR: 0.27, with 95% CI [0.16, 0.46], *p* < 0.001, I^2^ = 0%) (Figure 5A). Specifically, ivermectin significantly reduced the risk of a burning or stinging sensation (RR: 0.07, with 95% CI [0.02, 0.20], *p* < 0.001, I^2^ = 0%) (Figure 5B). However, there was no significant difference in the incidence of gastrointestinal adverse events between the two groups, although a trend toward a higher risk with ivermectin was noted (RR: 1.47, with 95% CI [0.67, 3.22], *p* = 0.34, I^2^ = 0%) (Figure 5C).

## Discussion

This systematic review and meta-analysis of 10 RCTs, encompassing 1,105 patients, demonstrated that ivermectin and benzyl benzoate have comparable efficacy in curing scabies across all follow-up periods. However, ivermectin showed a superior safety and tolerability profile. Oral ivermectin patients had fewer adverse events than topical benzyl benzoate patients, primarily due to reduced skin burning or irritation. Furthermore, there was no difference regarding adverse gastrointestinal events, despite the oral administration of ivermectin. The observation of similar efficacy between oral ivermectin and topical benzyl benzoate possesses considerable clinical significance, as clinicians are currently equipped with two potent therapeutic options. This is especially vital at present as there are increasing reports of treatment failure with 5% permethrin, which is a widely recommended first-line agent, and there have been increasing concerns about resistance, showing the need for strong alternatives (25, 26).

Nevertheless, this pooled efficacy estimate should be interpreted while considering substantial heterogeneity in treatment protocols across studies. Oral ivermectin regimens varied from one to two oral doses. Since ivermectin is not effective against mite eggs, a follow-up dose roughly 1 week after the initial treatment is typically recommended to target newly hatched mites and guarantee a full cure (1). Several included trials utilized only a single dose of ivermectin (6, 10, 11, 22, 24), a protocol that likely diminishes the drug’s true potential efficacy. Similarly, the benzyl benzoate treatments varied considerably, with concentrations spanning 10–25%, application frequencies ranging from a single application to five nights in a row, and contact times from 8 to 72 h. The considerable absence of a standardized treatment protocol for benzyl benzoate complicates direct comparisons and hinders the determination of an optimal topical regimen. Our subgroup analysis provides further clarity on this issue. When stratified by benzyl benzoate concentration, oral ivermectin demonstrated clear superiority at concentrations below 25%, with significant improvement in pruritus compared to benzyl benzoate (4, 11). However, when the comparator concentration reached 25% or higher, the advantage of oral ivermectin was no longer statistically significant (12, 23).

Regarding pruritus improvement, the analysis indicated a trend toward symptom alleviation with ivermectin, although statistical significance was not attained. Pruritus is known to potentially persist for weeks despite successful mite eradication as a result of a prolonged hypersensitivity reaction to mite antigens and feces retained in the epidermis (27). This phenomenon may clarify the less definitive nature of this outcome in comparison to clinical/parasitological cure (28). Accordingly, ivermectin also exhibits inherent anti-inflammatory characteristics, a mechanism that explains its effectiveness in the treatment of inflammatory dermatoses such as rosacea (29–31). This anti-inflammatory activity may contribute to a quicker resolution of scabies-related pruritus, suggesting a dual mechanism of action requiring further study (30).

Moreover, oral ivermectin’s superior safety profile is a key finding. The near elimination of local skin irritation was the primary reason for this well-tolerability. Ivermectin was associated with a 93% relative risk reduction in burning and stinging sensations, which is a major clinical advantage. Local irritation from benzyl benzoate is a recognized side effect and a key factor in patients not adhering to their treatment, notably in pediatric patients, pre-existing dermatitis patients, or patients with skin damage from excessive scratching (32). Poor compliance reduces its real-world effectiveness since incomplete or incorrect application may cause treatment failure (33). Ivermectin’s excellent tolerability, achieved by avoiding topical application, not only improves patient comfort but also directly enhances its effectiveness in clinical practice (33).

However, the analysis revealed a non-significant trend that suggested that using ivermectin may slightly increase the risk of mild gastrointestinal adverse events, such as nausea. It is important to consider that these events were rare in the included trials and are generally reported as mild and temporary in the wide literature (34). Therefore, even with this small trend, ivermectin is still much safer and better tolerated than topical benzyl benzoate, which has significant local side effects.

Furthermore, this pooled analysis expands on previous systematic reviews, including Strong and Johnstone’s (35) Cochrane review, which highlighted a lack of robust data to definitively compare the effectiveness of numerous scabicides, thus emphasizing the importance of this targeted meta-analysis. Furthermore, our findings are highly relevant to the evolution of clinical practice guidelines. Major international societies, including the World Health Organization (WHO) and the European Academy of Dermatology and Venereology, recommend both ivermectin and benzyl benzoate as effective treatments for scabies (1, 36, 37). The simplicity of oral ivermectin administration—usually a single dose repeated once—is a major benefit over the frequently difficult, time-consuming, and messy benzyl benzoate application. The tolerability of treatment is critical to its effectiveness in the real world, particularly when handling large families, mass drug administration programs, and widespread outbreaks.

### Limitations

This review has several important limitations. First, there is a considerable risk of bias in the included studies. The majority of trials had either some concerns or a high risk of bias, primarily due to their open-label design. This introduces a notable risk of performance and detection bias, especially for subjective outcomes such as clinical lesion assessments and patient-reported itching scores. Consequently, the certainty of evidence for outcomes was rated as ‘Low’ to ‘Moderate’ according to the GRADE framework. Second, substantial clinical heterogeneity in treatment protocols is a key limitation. The wide variation in benzyl benzoate regimens—including concentration, frequency of application, and duration of contact—as well as differences in ivermectin dosing strategies (e.g., single vs. double dose), may obscure or dilute true differences between standardized regimens.

Third, inconsistency in outcome definitions across studies may affect the reliability of pooled estimates. Key outcomes such as “cure rate” ranged from strict parasitological confirmation to purely clinical assessments, while definitions of “pruritus improvement” also varied considerably. Additionally, the lack of homogeneity in the age ranges of the study populations limits the generalizability of findings. Some studies were conducted in children, while others were conducted in young or older adults, introducing variability in treatment responses and outcome measures that may impact the comparability of results across trials. These limitations highlight the need for more rigorous, standardized, and age-consistent trials to strengthen the evidence base.

### Implications for future research

Accordingly, high-quality, large-scale, double-blinded RCTs should be given priority in future research to address the shortcomings of the current body of evidence. Standard validated outcome measures, such as patient-reported outcomes for symptoms like pruritus and objective parasitological confirmation of cure rate (e.g., dermoscopy or skin scraping), should be used in these trials. Additionally, studies that directly compare optimal treatment plans—for example, comparing a two-dose oral ivermectin strategy to a standardized, evidence-based benzyl benzoate strategy—are desperately needed. Finally, to better assess relapse and recurrence rates, future research should adopt longer follow-up periods (such as longer than 3 months), and formal economic analyses should be incorporated to compare the cost-effectiveness of various treatment approaches in diverse settings.

## Conclusion

This systematic review and meta-analysis show that oral ivermectin and topical benzyl benzoate offer comparable efficacy for the treatment of scabies. However, oral ivermectin has demonstrated a better safety and tolerability profile, combined with the profound practical advantages of its simple oral administration, establishing it as a highly valuable and often preferable treatment option.

## Data Availability

The original contributions presented in the study are included in the article/Supplementary material, further inquiries can be directed to the corresponding author.
