# Supplementary material for: Efficacy and safety of oral ivermectin versus benzyl benzoate for the treatment of scabies: a systematic review and meta-analysis of randomized controlled trials
Source: Front Med (Lausanne). 2025 Nov 6;12:1703912. doi: 10.3389/fmed.2025.1703912 (PMC12629932; doi:10.3389/fmed.2025.1703912)
Supplement: Supplementary file 1 [file Table_1.docx]

**Table S1**. Search Strategy.

| **Database** | **Search Terms** | **Search Field** | **Search Results** |
| --- | --- | --- | --- |
| PubMed | (Scabies OR "Norwegian Itch" OR "Sarcoptes scabiei" OR “Sarcoptic Mange”) AND (ivermectin OR Eqvalan OR Ivomec OR MK-933 OR MK933 OR “MK 933” OR Mectizan OR Stromectol) AND ("benzyl benzoate" OR "BB" OR Benzanil OR Novoscabin OR Ansar OR Antiscabiosum OR Ascabiol OR Benzemul OR Acarosan OR Acaril) | All Fields | 75 |
| Cochrane | (Scabies OR "Norwegian Itch" OR "Sarcoptes scabiei" OR “Sarcoptic Mange”) AND (ivermectin OR Eqvalan OR Ivomec OR MK-933 OR MK933 OR “MK 933” OR Mectizan OR Stromectol) AND ("benzyl benzoate" OR "BB" OR Benzanil OR Novoscabin OR Ansar OR Antiscabiosum OR Ascabiol OR Benzemul OR Acarosan OR Acaril) | All Text | 17 |
| Web of Science | (Scabies OR "Norwegian Itch" OR "Sarcoptes scabiei" OR “Sarcoptic Mange”) AND (ivermectin OR Eqvalan OR Ivomec OR MK-933 OR MK933 OR “MK 933” OR Mectizan OR Stromectol) AND ("benzyl benzoate" OR "BB" OR Benzanil OR Novoscabin OR Ansar OR Antiscabiosum OR Ascabiol OR Benzemul OR Acarosan OR Acaril) | All Fields | 98 |
| Scopus | TITLE-ABS-KEY ( ( Scabies OR "Norwegian Itch" OR "Sarcoptes scabiei" OR "Sarcoptic Mange" ) AND ( ivermectin OR Eqvalan OR Ivomec OR MK-933 OR MK933 OR "MK 933" OR Mectizan OR Stromectol ) AND ( "benzyl benzoate" OR "BB" OR Benzanil OR Novoscabin OR Ansar OR Antiscabiosum OR Ascabiol OR Benzemul OR Acarosan OR Acaril ) ) | Title, Abstract, Keywords | 325 |
| Google Scholar | (Scabies OR "Norwegian Itch" OR "Sarcoptes scabiei" OR “Sarcoptic Mange”) AND (ivermectin OR Eqvalan OR Ivomec OR MK-933 OR MK933 OR “MK 933” OR Mectizan OR Stromectol) AND ("benzyl benzoate" OR "BB" OR Benzanil OR Novoscabin OR Ansar OR Antiscabiosum OR Ascabiol OR Benzemul OR Acarosan OR Acaril) | All Fields | 98 |

**Table S2**. Excluded records in full-text screening.

| **Title** | **Published Year** | **DOI** | **Study** | **Exclusion Reason** |
| --- | --- | --- | --- | --- |
| Comparison of topical permethrin 5% vs. benzyl benzoate 25% treatment in scabies: a double-blinded randomized controlled trial. | 2024 | 10.1093/bjd/ljad501 | Meyersburg 2024 | Wrong intervention |
| Ivermectin alone or in combination with benzyl benzoate in the treatment of human immunodeficiency virus-associated scabies. | 2000 | 10.1046/j.1365-2133.2000. 03480.x | Alberici 2000 | Wrong comparator |
| Comparison of the effects of ivermectin, permethrin, and gamma benzene hexachloride alone and with that of combination therapy for the management of scabies | 2022 | 10.47750/jptcp.2022.845 | Dey 2022 | Wrong intervention; |
| Assessment of different treatment options for scabies | 2023 | - | Verma 2023 | Wrong study design |

**Table S3**. Subgroup analyses of efficacy outcomes comparing oral ivermectin (single vs. double dose) with topical benzyl benzoate.

| **Outcome** | **Time point** | **Dose** | **Number of studies** | **RR** | **95% CI** | **I^2^** | **P value for significance** |
| --- | --- | --- | --- | --- | --- | --- | --- |
| Cure rate | 1 week | Single dose | 5 | 1.09 | (0.84, 1.42) | 46% | 0.5 |
|  |  | Double dose | 1 | 0.92 | (0.60, 1.40) | - | 0.69 |
| Cure rate | 2-4 week | Single dose | 5 | 1.03 | (0.73, 1.45) | 89% | 0.88 |
|  |  | Double dose | 3 | 1.09 | (0.89, 1.34) | 66% | 0.39 |
| Pruritic improvement | 2-4 week | Single dose | 2 | 1.27 | (1.04, 1.56) | 5% | 0.02 |
|  |  | Double dose | 2 | 1.36 | (1.01, 1.83) | 36% | 0.04 |

CI: confidence interval; RR: risk ratio

**Table S4**. Subgroup analyses of efficacy outcomes comparing oral ivermectin with different concentrations of topical benzyl benzoate (<25% vs. ≥25%)

| **Outcome** | **Time point** | **Dose** | **Number of studies** | **RR** | **95% CI** | **I^2^** | **P value for significance** |
| --- | --- | --- | --- | --- | --- | --- | --- |
| Cure rate | 1 week | <25% | 1 | 0.92 | (0.60, 1.40) | - | 0.69 |
|  |  | ≥25% | 5 | 1.09 | (0.84, 1.42) | 46% | 0.5 |
| Cure rate | 2-4 week | <25% | 3 | 0.83 | (0.49, 1.43) | 91% | 0.5 |
|  |  | ≥25% | 5 | 1.15 | (0.99, 1.35) | 67% | 0.07 |
| Pruritic improvement | 2-4 week | <25% | 2 | 1.29 | (1.05, 1.58) | 0% | 0.02 |
|  |  | ≥25% | 2 | 1.35 | (0.99, 1.86) | 45% | 0.06 |

CI: confidence interval; RR: risk ratio
